# RTMS for negative symptoms of schizophrenia efficacy and safety of high-frequency repetitive transcranial magnetic stimulation with H7-coil for negative symptoms of schizophrenia

**DOI:** 10.1192/j.eurpsy.2026.10351

**Published:** 2026-07-31

**Authors:** Igor Filipčić, Katarina Matić, Ivona Šimunović Filipčić

**Affiliations:** 1 Psychiatric Clinic Sveti Ivan Zagreb, Croatia; 2 School of Medicine, University of Zagreb, Zagreb, Croatia; 3 Department of psychiatry and psychological medicine, University Hospital Center Zagreb, Zagreb, Croatia

## Abstract

**Objectives:**

To evaluate the efficacy and safety of high-frequency repetitive transcranial magnetic stimulation (HF rTMS) using the H7-coil, delivered once daily over 20 consecutive weekdays, as an adjunct to stable antipsychotic treatment for persistent negative symptoms in schizophrenia, a randomized, sham-controlled trial.

**Methods:**

We conducted an industry-independent, randomised, double-blind, sham-controlled superiority trial with concealed allocation and masked outcome assessment, between 2019 and 2025, at the Sveti Ivan Psychiatric Hospital in Zagreb, Croatia. Eligible participants were outpatients diagnosed with schizophrenia (ICD-11: F20), aged 18–55 years, of both sexes, with a PANSS negative symptom subscale score > 24 and positive subscale score < 20, and stable antipsychotic therapy for ≥3 months. The primary outcome was the change in total score on the Scale for the Assessment of Negative Symptoms (SANS), adjusted for key covariates. Bayesian modelling was employed throughout, estimating treatment effects via posterior probabilities and Bayes factors (BFs).

**Results:**

A total of 87 participants were randomised: 43 to the H7-coil arm (34% female) and 44 to the sham arm (32% female). Median age was 38 (IQR 27–46) years in the H7 group and 34 (IQR 26–45) years in the sham group. The groups were well balanced across key prognostic variables. Both groups showed improvement in negative symptoms over time. In the H7-coil arm, the adjusted median reduction in SANS total score was 37 points (41%), compared to 26 points (31%) in the sham group. Bayesian analysis yielded a posterior probability of 0.87 that the H7-coil was superior to sham (β < 0), with a corresponding BF of 3.4, indicating moderate evidence in favour of the H7-coil. Evidence of superiority was also observed for the SANS subdomains of blunted affect, alogia, and avolition (BFs 3.0–5.6), as well as for the BNSS total score. No serious adverse events were reported.

**Conclusion:**

This trial provides bayesian evidence that HF rTMS with the H7-coil may reduce negative symptom severity in schizophrenia and appears to be safe and well tolerated. These findings support further investigation of deep rTMS as an adjunctive treatment for persistent negative symptoms.

**Keywords:**

TMS; Schizophrenia; Psychotic disorder; negative symptoms; H7-coil; sham-coil.

**Disclosure of Interest:**

None Declared

